# Tuberculosis of the Penis: A Review of the Literature

**DOI:** 10.1155/2015/601624

**Published:** 2015-09-08

**Authors:** Anthony Kodzo-Grey Venyo

**Affiliations:** Department of Urology, North Manchester General Hospital, Delaunay's Road, Manchester, UK

## Abstract

*Background*. Tuberculosis of the penis (TBP) is rare.* Aim*. To review the literature.* Method*. Various internet data bases were searched.* Literature Review*. TBP could be primary or secondary, may develop following circumcision performed by a person who had pulmonary Tb, and may be transmitted to the penis from ejaculation, contamination from clothing, or from contact with endometrial secretions, following an earlier pulmonary Tb or Tb elsewhere. TBP presents with a painless/painful small nodule, ulcer, mass on penis which gradually enlarges, and induration/swelling of penis, with or without erectile dysfunction. Inguinal lymph nodes may or may not be palpable. The patient's voiding is normal. There may or may not be history of circumcision, pulmonary Tb, and BCG immunization. TBP mimics penile carcinoma, granulomatous syphilis penile ulcer, genital herpes simplex, granuloma inguinale, and HIV infection. Diagnosis is established by microscopic examination finding of granulomas +/−AFB in penile discharge or biopsy of lesion or culture of Tb organism from discharge or biopsy specimens or positive Elisa serology/PCR for Tb. PTBs respond to first- or 2nd-line anti-Tb 6-month treatment. Close contacts should be screened. Extrapulmonary Tb should be excluded. *Conclusions*. Clinicians should consider possibility of PTB in cases of penile lesions and erectile failure.

## 1. Introduction

Tuberculosis [Tb] is the most widespread and persistent human infection in the world. The infection can affect any organ and mimic other illnesses; hence it is called the great mimicker [1–3]. Globally, no country has ever been able to eradicate Tb [2, 4]. Tb of the penis is an uncommon presentation of Tb, even in countries where the incidence of pulmonary and extrapulmonary Tb is high. Even in Tb endemic countries it is difficult to diagnose Tb of the penis. It would even be more difficult to diagnose Tb of the penis in Tb non-endemic areas of the world because a number of clinicians would be unaware of the possibility of the occurrence of such a rare disease as well as the clinical manifestation of the disease. The ensuing review paper on Tb of the penis has been divided into two parts: (A) overview and (B) miscellaneous narrations and discussions from some reported cases.

## 2. Aim

The aim of this study is to review the literature on tuberculosis of the penis.

## 3. Method

Various internet data bases were searched including the following: PUB Med, Google, Google scholar, and Educus. The following search words were used: tuberculosis of the penis and penile tuberculosis. Information obtained from 44 references was used to write the literature review.

## 4. Literature Review

### 4.1. Overview

#### 4.1.1. General Comment

- Tb of the penis may either be primary Tb or secondary Tb but on the whole most of the reported cases have been primary Tb.

#### 4.1.2. Presentation

Tb of the penis may present as one of the following:

- Small ulcer on glans penis/corona (painless or painful).
- Multiple small ulcers on the penis.
- A gradually enlarging ulcer on the glans penis.
- A nodule on the penis.
- An induration of the penis.
- A swelling of the penis.
- Erectile failure.
- When usually there is no urinary tract symptom.
- In cases of secondary Tb where there may be a history of pulmonary Tb.
- Discharge from ulcer on penis.
- A mass/lump on glans penis/prepuce covering the external urethral meatus mimicking carcinoma.

#### 4.1.3. Clinical Examination Findings

The findings of the clinical examination are as follows:

- Ulcer on dorsal aspect of glans penis and/or prepuce.
- Swollen penis.
- Nodule on penis.
- Fungating growth on penis without involvement of urethra.
- The inguinal lymph nodes which may or may not be palpable.

#### 4.1.4. Investigations

*(1) Haematological Investigations*

- In full blood count there may or may not be lymphocytosis and the erythrocyte sedimentation rate (ESR) may or may not be raised.

*(2) Biochemical Tests*

- Serum urea and electrolytes and liver function tests as well as serum glucose should be normal unless the patient has any other disease that may impair the results the tests.

*(3) Urinalysis, Urine Microscopy, and Culture*

- Urinalysis and urine culture and sensitivity tests should be normal and these usually should show no growth of a Gram positive or Gram negative organism (nevertheless in cases that may be associated with Tb of the kidney and the genitourinary tract there could be sterile pyuria and perhaps positive tuberculosis culture related to the genitourinary tract Tb but not the penile Tb).

*(4) Mantoux Test*

- Mantoux test may be strongly positive but at times it may be negative.

*(5) Elisa Serology/Polymerase Chain Reaction (PCR) Tests*

- Elisa serology/PCR tests when done would be positive for Tb in cases of Tb of penis.

*(6) Bacteriological Microscopy, Culture, and Sensitivity*

- Direct microscopic examination of discharge from the penile ulcer/growth may reveal granulomas and mycobacterium.
- Culture and sensitivity tests of discharge from the penile lesion or biopsy for acid fast bacillus may confirm Tb and confirm the sensitivity pattern.

*(7) Radiological Images*

*(a) Chest X-Ray*

- Chest X-ray can be done to confirm absence or presence of pulmonary Tb in patients affected by Tb of penis or in their contacts who are being screened.

*(b) Ultrasound Scan of Abdomen, Pelvis, Renal Tract, and Scrotal Contents*

- Ultrasound scan may indicate absence or presence of Tb in the genitourinary tract including the kidney, testis, epididymis, and seminal vesicles of the patients and also their contacts and family and in females, presence or absence of abnormality of the endometrium may be seen.
- The scan may also confirm or exclude presence or absence of enlarged lymph nodes in the pelvis and inguinal region.

*(c) Computed Tomography (CT) Scan*. CT scan of abdomen, pelvis, and thorax when undertaken may confirm or exclude presence or absence of pulmonary Tb in the patient, his contacts, and family. CT scan would also confirm or exclude presence of extrapulmonary Tb or enlarged inguinal lymph nodes.

*(d) Magnetic Resonance Imaging (MRI) Scan*. MRI scan of abdomen, pelvis, and thorax when undertaken may confirm or exclude presence or absence of pulmonary Tb in the patient, his contacts, and family. MRI scan would also confirm or exclude presence of extrapulmonary Tb or enlarged inguinal lymph nodes.

*(8) Macroscopic Findings*. Some of the possible findings on gross examination of the penile lesion before and after biopsy include the following:

- An ulcerated foul smelling necrotic area on the glans penis with an indurated irregular edge and the base of the ulcer may be granular with a serosanguinous discharge.
- Firm cauliflower growth with nodular surface which is seen to have occupied the entire glans penis.
- Multiple superficial ulcers on the prepuce and glans penis. The edges of the ulcers may be undermined.

*(9) Histopathological Findings*. Histological examination of biopsy specimens of the penile lesion in Tb of penis may show the following:

- Caseating granulomas without any cause identified.
- Caseating granulomas and acid fast bacillus.

*(10) Differential Diagnoses*. Some of the differential diagnoses include the following:

- Carcinoma of penis.
- Granulomatous syphilis ulcer of penis.
- Primary Tb of the penis is an extremely rare condition, should be differentiated from other venereal diseases apart from syphilis, some of which include the following three diseases:
  - Genital herpes simplex.
  - Granuloma inguinale.
  - Human immunodeficiency virus (HIV) infection.

*(11) Treatment*

- First-line anti-Tb treatment tends to be effective in the treatment of Tb of penis but in cases of multiresistant Tb infections of the penis second-line anti-Tb treatment should be effective. Considering the fact that multiresistant Tb could affect the penis, in positive Tb cultures sensitivity patterns should be obtained to guide the clinician regarding the correct treatment regime.
- Usually six months of treatment should be adequate and whenever possible a second biopsy from the site of Tb of penis should be taken for histological examination to confirm absence of Tb.
- The family and close contacts of a patient with Tb of penis should be screened and those found to have Tb should be treated appropriately for Tb.

*(12) Outcome*

- Tb of the penis usually responds to six months of first-line anti-Tb treatment but patients with multiresistant Tb of the penis would require 2nd-line anti-Tb treatment which should be effective.

### 4.2. Miscellaneous Narrations and Discussions from Some Reported Cases

#### 4.2.1. Miscellaneous Narrations (See Table 1 Which Contains a Summary of Some of the Reported Cases)

Pal et al. [5] in 1996 reported 2 cases of primary tuberculosis of penis as follows:

**Case 1 .**

**Figure 1:**
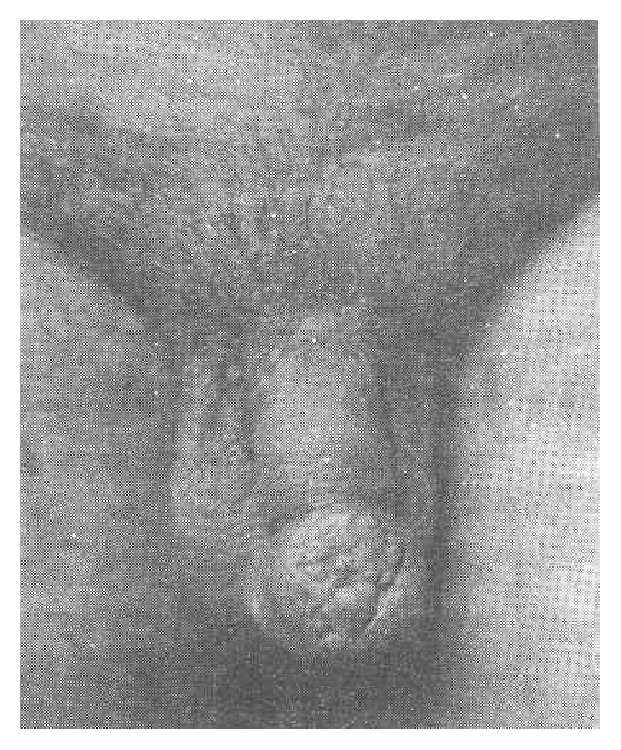
Showing tuberculosis of the glans penis. Reproduced from [5] D. K. Pal, A. K. Kundu, S. Chakraborty, S. Das, “Tuberculosis of Penis: Report of Two Cases,” Ind. J Tub vol. 43; pp. 203-204, 1996. Copy right Indian Journal of Tuberculosis. Reproduced with permission granted by the editor of the journal, who stated that permission to make a copy of the paper has been granted subject to the following: (1) the paper should only be used for academic and research purposes and not for profit/business. (2) Permission is given with the proviso that the Indian Journal of Tuberculosis is cited as the source of the figure. The original copy right is retained by Indian Journal of Tuberculosis and any request to use or reproduce the figure would require permission from the Indian Journal of Tuberculosis.

A 24-year-old unmarried man presented with a slowly enlarging swelling of the glans penis which he had had for six months prior to his presentation. The penile lesion originally started as a small ulcer on the dorsal aspect of the glans penis which slowly and progressively enlarged and this was ensued by the development of multiple variably sized nodules on the ulcerated glans penis. He did not have any problem with voiding. On clinical examination he was found to have a tender and firm cauliflower growth with nodular surface which had occupied the entire glans penis (see Figure 1). The growth had encompassed the external urethral meatus which was hidden beneath the growth. There were no palpable inguinal lymph nodes. Clinically the lesion was described as mimicking carcinoma of penis. His investigations were reported as follows:

- Haemoglobin was 10.9 g%, TLC was 13000/cu mm, N was 65, L was 31, M was 06, B was 00, and ESR was 46 mm/hour.
- Renal function tests: normal.
- Chest X-ray: normal.
- Urine for acid fast bacillus × 3: negative on each occasion.
- Intravenous urography: normal.

He had biopsy of the penile growth under local anaesthesia of which histological examination was reported as having shown tuberculous balanitis. Pal et al. [5] stated that up to 1971, 171 cases of Tb of the penis had been reported in the literature [11].

**Case 2 .**

**Figure 2:**
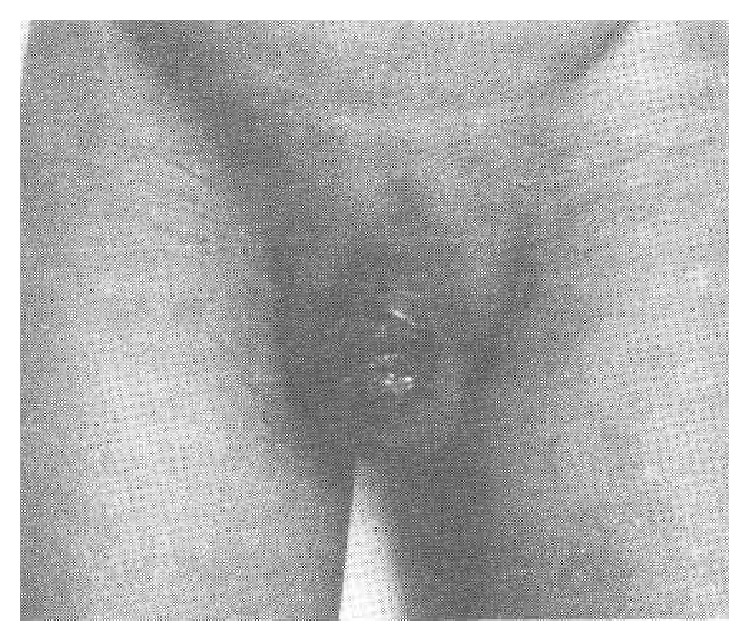
Showing tuberculous ulcer of glans penis. Reproduced from [5] D. K. Pal, A. K. Kundu, S. Chakraborty, S. Das, “Tuberculosis of Penis: Report of Two Cases” Ind. J Tub vol. 43, 203-204, 1996. Copy right Indian Journal of Tuberculosis. Reproduced with permission granted by the editor of the journal, who stated that permission to make a copy of the paper has been granted subject to the following: (1) the paper should only be used for academic and research purposes and not for profit/business. (2) Permission is given with the proviso that the Indian Journal of Tuberculosis is cited as the source of the figure. The original copy right is retained by Indian Journal of Tuberculosis and any request to use or reproduce the figure would require permission from the Indian Journal of Tuberculosis.

A 30-year-old married man reported an ulcer on the dorsal surface of his glans penis which had been present for 3 months (see Figure 2). He did not have any urinary symptoms. He gave a history of having had extramarital exposure 12 years earlier. On examination, an ulcerated foul smelling necrotic area was found on his glans penis with an indurated irregular edge and the base of the ulcer was granular with a serosanguinous discharge. He had bilateral inguinal lymphadenopathy. His prostate, seminal vesicles, testes, and epididymis on both sides were felt to be normal on examination. A provisional diagnosis of granulomatous syphilitic ulcer of penis was made. His investigations were reported as follows:

- Haemoglobin was 11.2 g%, TLC was 13800/cu mm, N was 68%, I^∧^ was 36%, E was 4%, M was 2%, B was 0%, and ESR was 36 mm/hour.
- Serum urea and electrolytes as well as blood sugar were normal.
- Repeated VDRL tests were negative (excluding syphilis).
- Urine microscopy was normal.
- Chest X-ray was normal.
- Three consecutive urine tests for acid fast bacilli were negative.
- Intravenous urography was normal.
- Mantoux test reading after 72 hours did not show any induration.

He had biopsy of the lesion under local anaesthesia and histological examination of the biopsy specimen showed tuberculous granuloma with intense fibrosis and endarteritis.

With regard to management of the two cases, both patients were treated with rifampicin, pyrazinamide, ethambutol, and isoniazid (INH). After two months, pyrazinamide and ethambutol were stopped but the remaining two antitubercular drugs were continued for 7 months. After 9 months of chemotherapy, the first patient underwent circumcision and histological examination of the preputial skin showed that there was no evidence of residual disease. With regard to the second patient, his penile ulcer had healed after 3 months of antituberculosis therapy. Unfortunately, the patient was lost to follow-up after his 7-month follow-up.

J. K. Kar and M. Kar [6] reported a 31-years-old man who presented with some ulcerated lesions on his glans penis and who had a strongly positive Mantoux test and positive TB-PCR. He had biopsy of the lesion and histological examination of the specimen showed features consistent with tuberculosis and a diagnosis of primary tuberculosis of the glans penis was made. There was no evidence of any coexisting tuberculosis anywhere else. The patient responded well to antitubercular therapy.

Baveja et al. [7] reported a 62-year-old man who had worked in a tuberculosis laboratory for 25 years in India preceding his presentation and who presented with multiple ulcerations on his glans penis for the preceding two years. In the first instance he had noticed a small painful raised lesion, which ulcerated and had started to discharge pus, but he did not have any other local or systemic symptoms. He was a widower and stated that he had not had any sexual partner for 10 years. His clinical examination revealed multiple superficial ulcers on the prepuce and glans penis. The edges of the ulcers were undermined; however, the ulcers did not perforate deeply into the urethra. He did not have any palpable inguinal lymph nodes. His general and systematic examinations were normal. His investigations were reported as follows:

- Erythrocyte sedimentation rate (ESR) was 44 mm/hour (raised).
- Venereal disease research laboratory (VDRL) and human immunodeficiency virus serology were negative.
- Chest X-ray was normal.
- Ultrasound scan of abdomen was normal.
- Urine microscopy and culture were normal and there was no growth of any organism.
- Direct smear microscopy of the pus showed a heavy growth of acid fast bacilli (3+).
- ELISA serology for mycobacterial A60-antigen was strongly positive (840 u/dL).

The patient was treated by means of first-line antitubercular treatment, without any clinical evidence of healing of his ulcers. In view of this cultures for* Mycobacterium tuberculosis* were set up on Lowenstein Jenson medium and in vials of BACTEC 12B liquid medium. Direct microscopy of the pus was done and this again revealed a heavy growth of acid fast bacilli (3+), and cultures from both BACTEC liquid medium and Lowenstein Jensen medium were positive for* Mycobacterium tuberculosis*. Routine bacterial culture did not have a growth of any other bacteria. Drug sensitivity tests were undertaken with the BACTEC 460 TB system which revealed resistance to the first-line drugs rifampicin and isoniazid. He was next treated with a second line antitubercular drug regimen according to the Revised National TB Control Programme protocol for multidrug resistant TB. After 3 months his ulcers had started to heal.

Angus et al. [8] reported two cases as follows.

**Case 1 .**

A 50-year-old Indian man who had returned to the United Kingdom from India was referred by his general practitioner because of a painless ulcer on his penis for two months. He noticed a small painless lump on his penis shortly after he had returned from India. The lump gradually increased in size. He was married and denied having had any other sexual partner for more than 25 years. He had lived in the United Kingdom for more than 40 years. He was found on examination to have a 1 cm diameter indurated ulcer which was also 1 cm in depth adjacent to the corona. The ulcer was nontender and had a rolled edge. He also had palpable small, mobile, nontender left inguinal nodes. He had a BCG scar on his arm. He had punch biopsy of the lesion and histological examination of the specimen revealed granulomas without any obvious cause identified. A multitude of stains done on the specimen yielded negative results and these include Gram stain, Ziehl-Neelsen, Giemsa, and fungal stains. The serum rapid plasma regain test and Treponema Pallidum Haemagglutination assay were reported as having yielded negative results. He had other investigations which were reported as follows:

- Three early morning specimens of urine were negative for mycobacteria on microscopy and culture.
- Cytological tests were negative.
- Chest X-ray was normal.
- Ultrasound scan of abdomen was normal.

An excision biopsy of the lesion was then undertaken and histological examination of the specimen showed caseating granulomas without any cause identified. All microbiology stains of the specimen were negative. Culture of tissue from the specimen yielded* Mycobacterium tuberculosis* which was susceptible to isoniazid, rifampicin, pyrazinamide, streptomycin, and ethambutol. He received antitubercular treatment for 6 months. The excision biopsy site healed without any recurrence of the penile lesion. The patient's family was screened for tuberculosis and they were all found not to have had tuberculosis. Nevertheless, the patient's wife refused to be screened.

**Case 2 (wife of Case 1).**

A 49-year-old Indian woman was seen by a gynaecologist because of a 3-month history of menorrhagia, fever, sweats, and weight loss. She had lived in the United Kingdom for 14 years and did not remember having had tuberculosis in her life. Her husband had been treated one year earlier for tuberculosis of the penis. She had endometrial biopsy and histological examination of the specimen showed multiple caseating granulomas but no cause was identified. On examination she was found to have a whitish, odourless vaginal discharge with ulcerations on the cervix. She had a number of investigations which were reported as follows:

- Erythrocyte sedimentation rate was 3 mm/hour.
- C-reactive protein was less than 8 mg/L (normal range < 8 mg/L).
- HIV and hepatitis B tests were negative.
- Rapid Plasma Reagin Test and Treponema Pallidum Haemagglutination Assay were negative.
- Urine dipstick test was normal.
- Chest X-ray showed a calcified granuloma in the left apex but there was no active lesions seen.
- Ultrasound scan of abdomen was normal.
- Early morning urine samples and vaginal swabs were negative upon culture.
- Culture of her second biopsy specimen grew* Mycobacterium tuberculosis* which was susceptible to isoniazid, rifampicin, pyrazinamide, streptomycin, and ethambutol.

 Angus et al. [8] further reported that both of the tissue cultured samples from the two cases were subjected to further tests by the Public Health Mycobacterium Laboratory in Dulwich in the United Kingdom who had confirmed the identification and sensitivities of the organism. The laboratory reported that Restriction Fragment Length Polymorphism Analysis which was based upon the IS6110 insertion element had shown that the organisms from both Cases 1 and 2 were identical in that all 14 bands were similar. Angus et al. [8] stated that the technique had been shown by Wilson et al. [12] to be more discriminatory than spoligotyping in outbreak investigations

KishanChand et al. [9] reported a 59-year-old Nepalese man who presented with an ulcer on his glans penis of 4-month duration. It started as a small ulcer on the dorsal aspect of the glans penis which measured approximately 0.5 cm × 0.5 cm but over a two-month period the ulcer had increased in size and destroyed and distorted most of the glans penis and it became painful. He did not have any voiding symptoms or haematuria. Most of the glans penis on examination was found to have been destroyed and the residual glans penis was found to have an irregular ulcerated growth with everted edges at the lateral and inferior aspects. The margins were irregular and the external urethral opening was distorted. The lesion was tender and its edges and base were indurated. He had bilateral inguinal lymph node enlargements. The inguinal lymph nodes were mobile, firm, discrete, and nontender. His general and systematic examinations were otherwise normal. A provisional diagnosis of carcinoma of penis was made and the edge of the lesion was biopsied. Histological examination of the biopsy specimen was reported as suggestive of a chronic granulomatous lesion, possibly caseating tuberculous granulation tissue. He had further evaluating tests which were reported as follows:

- Mantoux test was strongly positive.
- His ESR was 94 mm/hour.
- Histology of his lymph node biopsies showed reactive lymph nodes.
- Immunohistochemistry of the tissues of the lesion demonstrated antibody complexes of tuberculous bacilli.
- PCR was done which had confirmed tuberculosis.
- Relevant other investigations were done which had excluded pulmonary and renal Tb.

The patient was commenced on antitubercular therapy using 4-drug regimen of rifampicin, isoniazid, pyrazinamide, and ethambutol. The ulcer had healed and the penile pain had improved over two months of therapy and he was discharged on 2-drug regimen for 4 months and it was found at follow-up that his ulcer had healed. KishanChand et al. [9] stated the following:

- Histopathological examination of the involved tissue should be the essential initial investigation.
- Tuberculosis infection can be confirmed by Polymerase Chain Reaction (PCR) [13].
- Intravenous urography should be undertaken to exclude urinary tract Tb.
- Chest X-ray should be performed to exclude pulmonary Tb.

## 5. Discussion

The global Tb statistics has been reported as follows: [14]

- Globally there were an estimated 9 million new cases of Tb in 2013.
- Globally, there were an estimated 1.5 million deaths and of these 1.14 million deaths were among HIV negative people. Additionally there were 360,000 deaths among HIV positive people.
- There were an estimated 3.3 million cases and 519,000 Tb deaths among women.
- There were an estimated 500,000 cases of Tb in children and 80,000 deaths. The estimated number of deaths among children excluded Tb deaths in HIV positive children, for which the estimates were not available.
- Reports in 2012 [15] indicated that more than 10 million children had been orphaned as a result of the deaths of their parents from Tb.
- In 2012, there were an estimated 170,000 deaths from multidrug resistant (MDR) Tb and 450,000 new cases of MDR Tb.

It has been stated that Tb is one of the world's deadliest diseases and that [16]

- one third of the world's population is infected with Tb,
- in 2013, 9 million people globally became sick with Tb disease and there were about 1.5 million Tb related deaths globally,
- Tb is a leading killer of people who are HIV infected.

In the United States a total of 9,582 Tb cases (a rate of 3.0 cases per 100,000 persons) were reported in 2013 [16]. Both the number of Tb cases that were reported and the cases rate in the United States of America had decreased and this represented a 3.6% and 4.3% decrease, respectively, in comparison with 2012 [16].

Even though Tb is globally common, only sporadic cases of Tb of the penis are reported globally. In view of this, it would be argued that most practitioners would be unfamiliar with the presentation, investigation, diagnosis, and management of this extremely rare disease. It has been stated that Tb of the penis comprises less than 1% of all cases of Tb of the genitalia in males and the sites that are commonly affected include the epididymis in 42% of cases, seminal vesicles in 23% of cases, prostate in 21% of cases, testis in 15% of cases, and vas deferens in 12% of cases [7, 17].

With regard to the occurrence of Tb of the penis, Devine and associates [18] stated that the disease tends to occur in adults and it may be primary Tb or secondary to pulmonary Tb.

With regard to site of involvement and presentation of Tb, it has been stated that:

- the disease may affect the skin, glans penis, or cavernous bodies and in majority of cases the disease manifests as a superficial ulcer on the glans or around the corona in view of the fact that these are the commonest parts of the penis that are rubbed during sexual contact or with contaminated clothing,
- occasionally the disease may manifest as a solid nodule,
- Destruction of the glans penis may occur and advanced cases of Tb of the penis may manifest with erectile dysfunction as a sequel of tuberculous cavernositis [19].

Fournier was stated to have described in 1848 the first case of a patient with Tb of penis who had multiple ulcers of the penis and regional lymphadenopathy [8, 20]. Lewis [20] in 1946 reviewed 110 cases of Tb of the penis and Sekhon et al. [11] in 1971 reviewed 29 cases of Tb of penis that had been reported between 1946 and 1971. From 1971 through 1999, further 16 cases of Tb of the penis had been reported by a number of authors [19, 21–33] in the literature. Since then other cases of Tb of the penis have been sporadically reported including the case reported by Angus et al. [8] in 2001, the case reported by Baveja et al. [7] in 2007, and the case reported by J. K. Kar and M. Kar [6] in 2012.

Konohana and associates [34] in 1992 reported the first case of a culture-positive penile tuberculosis lesion which was shown to be culture-positive for* Mycobacterium tuberculosis*. Apart from* Mycobacterium tuberculosis* being known for causing tuberculosis of the penis, other* Mycobacterium* species can cause tuberculosis of the penis. In 1984, de Caprariis et al. [35] reported* Mycobacterium avium-intracellulare* as being responsible for causing penile lesion and venereal transmission. Dahl et al. [36] in 1996 reported* Mycobacterium celatum* as being responsible for causing a penile mass.

Pal [27] in 1997 as well as Murali and Raja [30] in 1998 reported tuberculosis of the penis as having been responsible for the cause of erectile dysfunction. Richards and Angus [29] in 1998 reported sexual transmission in tuberculosis of penis. The aforementioned narrations in a paper published by Angus et al. [8] of the wife of a patient who was successfully treated for Tb of penis with antitubercular first-line therapy who did not have Tb anywhere else in his body and whose wife was diagnosed as having endometrial Tb one year later without any previous history Tb elsewhere would also be suggestive of sexual transmission aetiology.

It has been stated that tuberculosis of the penis has been documented to have arisen historically from sexual contact with infected partner or from contamination from infected clothing [8, 20]. In 1913, Holt [37] described tuberculosis acquired through ritual circumcision. Tuberculosis of the penis in the 19th century was reported frequently in Jewish infants in whom ritual circumcision had been undertaken [8, 37]. Angus et al. [8] stated the following:

- With regard to the history of Tb of the penis, the religious leader after performing circumcision would suck the bleeding penis.
- Generally, extrapulmonary Tb is not considered as being infectious; nevertheless, in view of the fact that transmission from one mucosa to another during coitus is at least a theoretical possibility and in view of the fact that it has been described by some authors [38, 39] in animal models they had advised their patient not to have intercourse until one month of therapy had been completed as had been previously suggested by Septowitz [40] in 1996 but they treated the patient with anti-Tb therapy for 6 months.
- The differential diagnoses of chronic ulcer of penis with granulomas include bacterial infections, fungal infections, parasitic infections, sarcoidosis, and foreign body reactions.
- Very few organisms are present in most forms of cutaneous Tb; the results of both staining and culture most often are negative with the exception of scrofuloderma and cutaneous military Tb and in an effort to overcome this problem, some people had successfully utilised PCR to detect* Mycobacterium tuberculosis* DNA in cutaneous lesions; they had used PCR techniques to show sexual transmission.

Pal et al. [5] in 1996 had stated that in order to differentiate Tb of penis from carcinoma of the penis histopathological examination of the lesion would be essential. Furthermore Pal et al. [5] also in 1996 suggested that intravenous urography should be undertaken to exclude upper renal tract Tb. Pal et al. [5] made the following additional statements:

- Tuberculosis of the of the penis occurs in adults and it could be either primary Tb or secondary Tb as stated by Devine et al. [18].
- Cases of primary Tb of penis occur as a complication of ritual circumcision during which the operator sucks the circumcised penis. Some of these operators had pulmonary Tb [18, 19].
- Sucking of the penis had been undertaken as a haemostatic styptic measure but after turn of the century, the act of sucking the penis at circumcision had been practically eliminated from the ritual and as a result tuberculosis of the penis is rarely seen these days.
- Primary Tb of the penis can emanate as a sequel of coital contact with the disease already present in the female genital tract or even from infected clothing as reported by some authors [25].
- It had been postulated that the tuberculous bacilli are inoculated into abrasions that had been caused by vigorous sexual act in view of the fact that the normal mucosa is highly resistant to tuberculosis [11].
- It had also been postulated that, at times, a Tb lesion of the penis may be caused by Tb reinoculation of the male partner through his own infected ejaculate since the vagina is particularly resistant to Tb [18].
- Secondary Tb of the penis can occur associated with evidence of active pulmonary Tb elsewhere. And at times concomitant diabetes mellitus leads to such an atypical manifestation of Tb [41].
- Tb of the penis may affect the skin, glans, or cavernous bodies. In the majority of cases of Tb of the penis, the lesion appears as a superficial ulcer on the glans or around the corona in view of the fact that it is the commonest part of the penis rubbed during sexual contact or with infected clothing [19].
- Occasionally Tb of penis may manifest as a solid nodule.
- Destruction of the glans may be caused by the process of Tb of the penis and advanced cases of Tb of the penis may manifest with erectile dysfunction as a result of tuberculous cavernositis.
- The female partner of a patient with Tb of the penis should be assessed for genital tuberculosis.

Ghorbani et al. [10] reported a 43-year-old man who had had kidney transplantation and who presented with painful ulceration on his glans penis. Examination of his penis revealed an ulcer on the penis which was clinically considered to resemble herpetic ulcer or ulcer due to carcinoma. He did not have any history or clinical signs suggestive of Tb. He received empirical treatment against herpetic, bacterial, and fungal infection without any improvement in his penile ulcer. He had biopsy of the penile lesion and histological examination and PCR test had revealed* Mycobacterium tuberculosis*. He was treated with anti-Tb therapy and the ulcer improved and healed and there was no evidence of recurrence over a two-year-old follow-up. Ghorbani et al. [10] stated that in patients who have had renal transplantation who develop ulcers, especially in a Tb endemic area, if empirical treatment does not lead to improvement in the ulcers then the patients should be assessed for Tb. Ghorbani et al. [10] stated the following:

- Extrapulmonary Tb is a complication of pulmonary Tb which is disseminated via haematogenous and lymphatic systems and may involve any organ.
- Immunosuppressed patients such as renal transplant recipients have an increased risk of developing primary or secondary Tb.
- Some authors had recommended that Tb should be ruled out in high risk patients who present with chronic ulcers [42–44].
- Goens et al. [43] had iterated that primary Tb of the penis is an extremely rare condition which should be differentiated from other venereal diseases including syphilis, genital herpes simplex, granuloma inguinale, and human immunodeficiency virus (HIV) infection.

A number of urology departments are no longer routinely carrying out intravenous urography procedures. Instead of IVU most urology and radiology departments now routinely carry out other procedures including the following: ultrasound scan of abdomen, renal tract and pelvis; Computed Tomography (CT) scan of abdomen and pelvis; and Magnetic Resonance Imaging (MRI) scan of abdomen and pelvis. Ultrasound scan, CT scan, or MRI scan could be used to exclude Tb anywhere else in the abdomen and pelvis. Chest X-ray, CT scan of thorax, and MRI scan of the thorax are also radiological investigations that can be used to exclude pulmonary Tb. A number of investigations would be useful in confirming the diagnosis of Tb as a cause of a penile lesion and some of these include the following: direct microscopy and routine culture as well as AFB culture using Lowenstein Jensen Medium of discharge from the penile ulcer; routine culture and AFB culture of biopsy specimen of the penile lesion; histopathological examination of biopsy specimen of the penile lesion; PCR test to confirm Tb. It would be important to treat every case of Tb of the penis using first-line anti-Tb therapy for a sufficiently long time to ensure there is complete and full treatment of the Tb with no evidence of any Tb organism within the penis. In this case usually a period of 6-month therapy would be enough in most cases. It is also important to note that multiresistant Tb does occur and that some patients who develop Tb of the penis may have multiresistant Tb and that such patients would need second-line anti-Tb treatment for multiresistant Tb treatment. Rebiopsy of penile area involved by Tb may be required for Tb culture and histopathological examination to confirm successful treatment of the Tb by showing there is no residual Tb organism in the previously involved penile area. In view of the fact that anecdotal reports had documented sexual transmission of Tb in some cases of Tb of penis, all sexual partners of the patient diagnosed should be screened to exclude pulmonary and genitourinary Tb and these patients should have curettings from the endometrium for microscopy and culture for Tb as well as for histopathology to exclude Tb of the endometrium. All family and close contacts of patients who are diagnosed with Tb of the penis should be screened fully to exclude Tb whether or not they have had BCG vaccination. When a patient is diagnosed to have Tb of penis, he should be advised to abstain from sex for a period of time until the possibility of him transmitting Tb to his partner is over. There is no consensus opinion on how long such patients should abstain from sex but so far 1 month of abstention has been sufficient in sporadic cases. Nevertheless, considering the fact that multidrug resistant Tb does exist and clinicians may not know initially whether or not a person with Tb of penis has a multiresistant Tb or not especially in cases diagnosed from evidence of direct microscopy evidence of Tb in microscopic examination of smears from penile discharges. It is the view of the author that patients with Tb of the penis should abstain from sex for a period of time longer than one month until there is confirmation of absence of Tb whilst also ensuring the sexual partner has been investigated and found not to have Tb. If the sexual partner of a patient afflicted by Tb of the penis is found to have Tb of the endometrium or Tb anywhere else then she should be treated also. It is also worth noting that some patients with Tb of penis may have negative microscopy and culture for Tb in specimens of discharge and biopsies of their penile discharges and also that histopathological examination of their penile lesions may only show evidence of chronic granulomatous lesions without evidence of the Tb organism, in such cases Elisa serology/PCR test for Tb would be helpful in excluding other differential diagnoses.

## 6. Conclusions

Clinicians should consider possibility of PTB in cases of penile lesions and erectile failure.

Family and close contacts of patients with PTB should be screened to exclude Tb.

Although microscopic examinations of penile discharges and biopsy specimens may show granuloma and evidence of tuberculous Bacillus in some cases of PTB and at times AFB cultures from penile discharges may grow AFB, a number of these examinations may show only granulomas without presence of AFB.

Elisa serology/PCR tests for Tb are very useful for the confirmation of the diagnosis of penile TB.

In cases when relatives or close contacts of patients refuse to be screened for Tb gentle encouragement and education would be required by means of a multidisciplinary team approach.

Because of global travel, PTB could be encountered in all parts of the world and not only in areas where cases of Tb are common.

## Figures and Tables

**Table 1:** List and summary of the clinical findings of some of the reported cases of tuberculosis of penis.

Reference, year	Age; Presentation	Diagnostic and Histological findings Findings	Treatment	Outcome
Pal et al. [5]; 1996	Case 1: 24 years; tender/firm, cauliflower growth with nodular surface on glans penis.	Histology of biopsy specimen showed features of tuberculous balanitis.	Anti-Tb treatment with rifampicin, pyrazinamide, ethambutol, and isoniazid + circumcision 9 months later.	Complete healing and no recurrence.
	Case 2: 39 years; ulcer on dorsum of glans penis with foul smelling necrotic area on glans and serosanguinous discharge.	Histology of biopsy specimen showed tuberculous granuloma with intense fibrosis and endarteritis.	Anti-Tb treatment with rifampicin, pyrazinamide, ethambutol, and isoniazid.	Ulcer healed after 3 months but patient was lost to follow-up at 7 months.

J. K. Kar and M. Kar [6]; 2012.	31 years; ulcer on glans penis.	Positive Mantoux test; positive Tb-PCR. Biopsy of ulcerated lesion histology showed Tb of penis.	Anti-Tb treatment.	Responded well and ulcer healed.

Baveja et al. [7]; 2007	62 years; multiple ulcers on glans penis; he had previously worked in Tb laboratory; Multiple superficial ulcers on glans penis with undermined edges.	Direct smear microscopy of pus showed heavy growth of acid fast bacillus (3+); ELISA serology for mycobacterium A60-antigen was strongly positive.	1st line anti-Tb treatment but patient's lesion did not improve therefore he received second-line anti-Tb treatment.	Ulcer healed after 3 months of being on 2nd-line anti-Tb treatment after failure of 1st-line treatment.

Angus et al. [8]; 2001	Case 1 (husband of Case 2) 50 years; painless ulcer on penis.	Excision biopsy of penile ulcer showed on histological examination caseating granuloma and tissue culture of the biopsy yielded *Mycobacterium tuberculosis*.	He was treated with isoniazid, rifampicin, streptomycin, and ethambutol anti-Tb therapy for 6 months.	Would healed well with no recurrence.
	Case 2 (wife of Case 1) 49 years; menorrhagia; ulcerations on cervix.	Endometrial biopsy on microscopic examination revealed caseating granuloma.	Anti-Tb treatment with isoniazid, pyrazinamide, streptomycin, and ethambutol.	Cured and she became asymptomatic.

KishanChand et al. [9]; 2009	59 years; ulcer on glans penis with everted edges distorting the external urethral meatus; tender bilateral inguinal lymph node enlargement.	Biopsy specimen of ulcer on microscopic examination showed chronic granuloma; Mantoux test was strongly positive; immunohistochemical staining demonstrated antibody complexes of tuberculous bacilli; PCR confirmed Tb.	He was treated with rifampicin, isoniazid, pyrazinamide, and ethambutol.	Ulcer healed.

Ghorbani et al. [10]; 2007.	43 years; kidney transplant recipient; painful ulcer on glans penis.	Biopsy of the ulcer and PCR revealed *Mycobacterium tuberculosis*.	Anti-Tb treatment.	Ulcer healed.
